# Cerebral Microbleeds Are Associated with Deep White Matter Hyperintensities, but Only in Hypertensive Patients

**DOI:** 10.1371/journal.pone.0091637

**Published:** 2014-03-13

**Authors:** Zhongbao Gao, Wei Wang, Zhenfu Wang, Xingli Zhao, Yanchang Shang, Yaner Guo, Mei Gong, Lijuan Yang, Xiaobing Shi, Xian Xu, Ningyu An, Weiping Wu

**Affiliations:** 1 Department of Geriatric Neurology, Chinese PLA General Hospital, Beijing, People’s Republic of China; 2 Department of Radiology, Chinese PLA General Hospital, Beijing, People’s Republic of China; University of Manchester, United Kingdom

## Abstract

Cerebral microbleeds (CMBs) and white matter hyperintensities (WMH) are the most common manifestations of small vessel disease, and often co-occur in patients with cerebral vascular disease. Hypertension is widely accepted as a risk factor for both CMBs and WMH. However, the effect of hypertension on the association between CMBs and WMH remains unclear. We hypothesized that the relationship between CMBs and WMH is determined by hypertension. One hundred forty-eight patients with acute cerebrovascular disease who were admitted to PLA general hospital in Beijing, China from February 2010 to May 2011 were recruited in this study. CMBs on T2*-weighted angiography (SWAN) were assessed using the Brain Observer Microbleed Rating Scale criteria. The severity of the WMH was separately assessed as either peri-ventricular hyperintensities (PVH) or deep white matter hyperintensities (DWMH). The association among CMBs and the severity of WMH, and hypertension were determined. CMBs were found in 65 (43.9%) patients. The frequency of CMBs was related to the severity of DWMH and PVH. CMBs were more frequently observed in patients with hypertension compared to patients without hypertension (51.3% vs. 20.0%, *p = *0.001). Hypertension was an independent risk factor for CMBs (odds ratio 5.239, *p = *0.001) and DWMH (odds ratio 2.373, *p = *0.040). Furthermore, the relationship between the presence of CMBs and the severity of DWMH was only found in patients with hypertension (r = 0.298, *p*<0.01). However, CMBs were associated with PVH independently of hypertension. This study demonstrated that hypertension determined the association between CMBs and DWMH.

## Introduction

Cerebral microbleeds (CMBs) are focal deposits of hemosiderin in the brain, which is caused by a previous leakage of blood from small vessels, and are related to bleeding-prone microangiopathy of different origins [1]. These hemosiderin deposits can be visualized as small, homogeneous, round foci of low signal intensity as assessed using the T2*-weighted gradient-recalled-echo (GRE) and T2*-weighted angiography (SWAN) sequence of magnetic resonance imaging (MRI) [2], [3], [4]. Microbleeds have been more frequently observed in patients with both ischemic and hemorrhagic stroke. The frequency of CMBs is between 4%–5% in a healthy population, and can increase to 50%–70% in patients with cerebrovascular disease [1]. Among several causative factors, hypertension is the most consistent predictor of microbleeds, with odds ratio averages of 2.3 and 3.9 across studies in patients with stroke and healthy adults, respectively [5].

White matter hyperintensities (WMH) is frequently observed on brain MRI scanning in elderly people and is significantly associated with age, silent stroke, and hypertension. WMH, CMBs and lacunar infarcts are increasingly regarded as typical manifestations of cerebral small vessel diseases. The correlation between microbleeds and the severity of WMH was found in patients with primary stroke, patients with recurrent stroke and healthy subjects without major cerebrovascular risk factors; however, differences between deep white matter hyperintensities (DWMH) and peri-ventricular hyperintensities (PVH) have not been adequately addressed in these studies [6], [7], [8], [9], [10]. Yamada and colleagues previously reported an association among the number of CMBs, severity of PVH, and severity of DWMH, but failed to observe the effect of hypertension on the relationship between CMBs and PVH and DWMH [11]. Case-controlled studies have identified hypertension as one of the most important risk factors for WMH and microbleeds [12], [13]; however, the effect of hypertension on the association of microbleeds and WMH remains unclear. We performed the present study to investigate the effect of hypertension on the association between CMBs and PVH and DWMH separately in elderly patients with acute cerebrovascular disease.

## Materials and Methods

### Participants

We retrospectively recruited 173 inpatients who were admitted to our hospital due to fist-ever acute cerebrovascular disease from February 2010 to May 2011. All of the study participants were over 60 years of age. Of these 173 individuals, eight patients who did not undergo a brain MRI due to their poor condition and 17 patients who were unable to undergo MRI due to pacemakers and other implants were excluded. Thus, 148 patients were enrolled in this study cohort. Of these patients, 8 patients were diagnosed with intracranial hemorrhage (ICH), 63 patients had acute ischemic stroke (IS), 20 patients were diagnosed with transient ischemic attack (TIA), and 57 patients with lacunar stroke. The patients’ clinical information was obtained by personal interview, and a physical examination was performed by a staff member.

All of the participants signed informed consent to participate in the study. The study design was approved by the Ethics Committee of PLA General Hospital.

### Vascular Risk Factors and Cerebral Vascular Disease

In this study, hypertension was defined as a systolic blood pressure (SBP) ≥140 mm Hg, diastolic blood pressure (DBP) ≥90 mm Hg, or a history of hypertension as reported by the subject or indicated by anti-hypertensive therapy. The duration of the hypertension was assessed by clinical history, and all hypertensive patients had suffered from hypertension for more than 10 years. Hyperlipidemia was defined as a total cholesterol ≥240 mg/dL or a low-density lipoprotein cholesterol ≥160 mg/dL and/or the use of a cholesterol-lowering medication. Diabetes mellitus was defined as a fasting blood glucose ≥126 mg/dL (7.0 mmol/L), random blood glucose level ≥200 mg/dL (11.1 mmol/L) or reported treatment for diabetes mellitus. A current smoker was defined as a person who smoked every day, and a current drinker was defined as a person who consumed alcohol more than 1 day per week.

Acute cerebrovascular disease subtypes (i.e., ischemic stroke, lacunar stroke, TIA and intracranial hemorrhage) were diagnosed on the basis of cranial CT and/or MRI findings together with medical history, and neurological signs and symptoms and physical examination results at admission. Lacunar stroke followed the criteria of lacunar mechanism according to the Trial of Org 10172 in Acute Stroke (TOAST) classification [14]. Ischemic stroke included other subtypes of TOAST, except for lacunar mechanism. TIA was defined as an episode, focal neurological symptoms with abrupt onset and rapid resolution lasting under 24 hours due to altered circulation to a limited region of the brain with or without a corresponding lesion on diffusion-weighted imaging (DWI). Transient episodes without focal symptoms, isolated vertigo, migraine equivalents, and transient global amnesia were excluded from the TIA diagnosis.

### Blood Pressure Measurements

With the participants seated, we measured BP over the brachial artery of bilateral arms after a 5 min rest to make sure that the difference in blood pressure was less than 10 mm Hg. Then, the BP was calculated as the average of 3 resting brachial artery pressure readings of left arm. The BP was measured using a mercury-column sphygmomanometer with an appropriately sized arm cuff. The systolic BP (SBP) and diastolic BP (DBP) was recorded at the first appearance and disappearance (phase V) of Korotkoff sounds, respectively.

### MRI Scanning Protocol

Cranial MRI was performed using a Signa HD 1.5 Tesla (T) MRI scanner from GE (Fairfield, CT, USA). Transverse spin-echo T1- fluid - attenuated inversion recovery sequence(T1WI-FLAIR) (repetition time [TR]1875 ms, echo time [TE] 24 ms, inversion time [TI] 750 ms, Field of view [FOV] 240 mm, Acquisition time 2 min, Spatial resolution 0.83×0.83×6 mm), T2 - weighted sequence (T2-WI) (TR 5500 ms, TE 126 ms, FOV 240 mm, Acquisition time 1 min 50 sec, Spatial resolution 0.68×0.68×6 mm), T2-FLAIR (TR 8400 ms, TE 120 ms, TI 2100 ms, FOV 240 mm, Acquisition time 2 min 32 sec, Spatial resolution 0.83×0.83×6 mm), and DWI (b = 0 and 1000 s/mm^2^, TR 7000 ms, TE 79 ms, FOV 240 mm, Acquisition time 28 sec, Spatial resolution 1.88×1.88×6 mm) sequences were acquired. All the images were two dimensions.

The SWAN sequences were acquired using a three-dimensional (3D)-enhanced T2*-weighted contrast flow-compensated multi-echo gradient echo sequence with the following parameters: TR 65.5 ms, TE 36 ms, flip angle 20 degrees, receiver bandwidth ±625, FOV 240 mm, spatial resolution 0.63×0.75×3 mm, Acquisition time 4 min 41 sec, 1 transverse slab with 40 slices a single spatial excitation, flow compensation, parallel imaging with an acceleration factor of 2 and gap of 2 mm.

### Image Analysis

**Figure 1:**
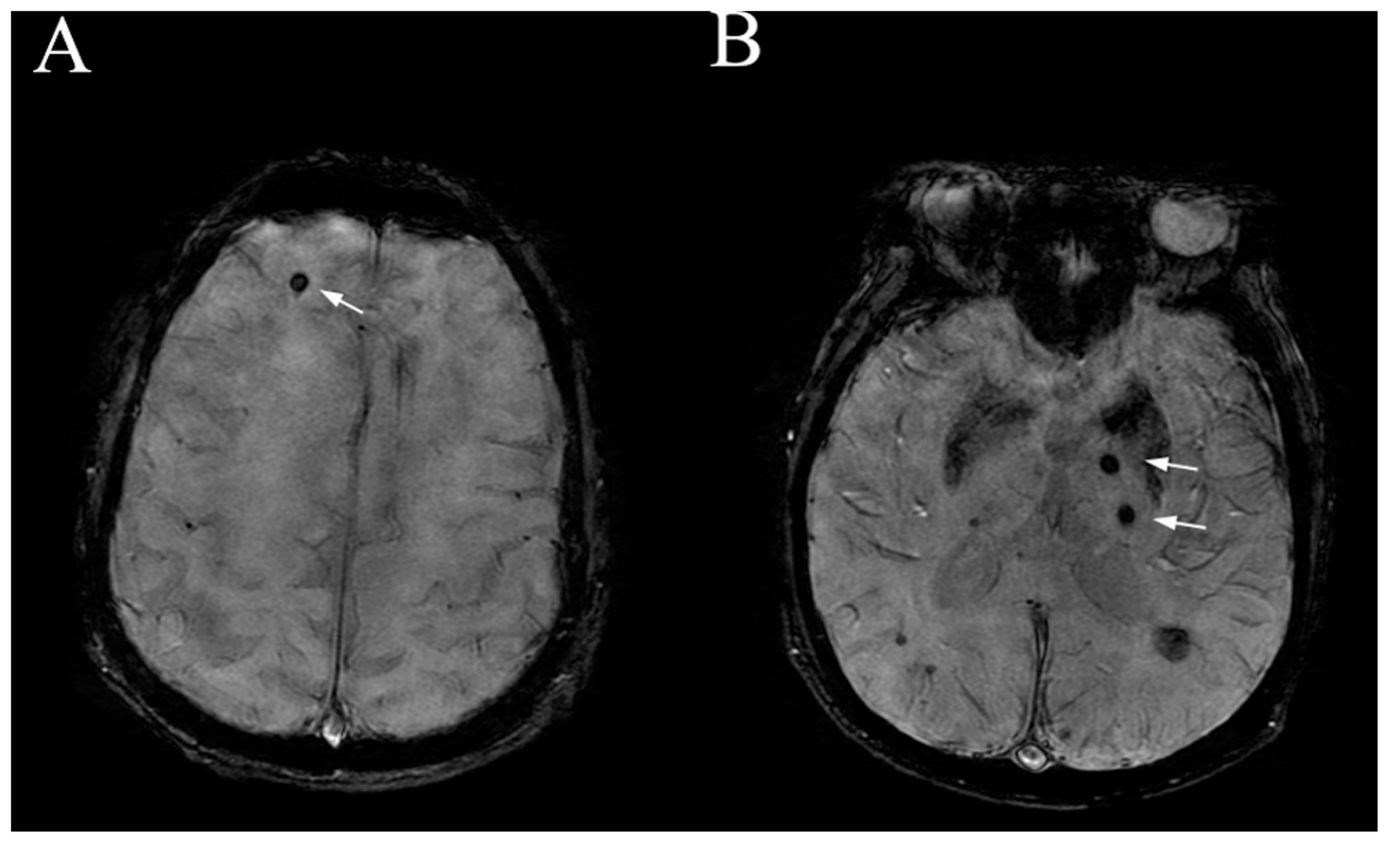
The location of cerebral microbleeds (CMBs) on 3D-enhanced T2*-weighted gradient echo sequence of MRI (arrows). (A) CMBs in frontal lobe. (B) CMBs in thalamus.

CMBs were defined as small foci with round hypointense signals that were 2 to 10 mm in diameter on the SWAN images (Fig. 1). CMBs were differentiated from areas of signal loss on the basis of vascular flow voids. Areas of symmetric hypointensity in the basal ganglia, which potentially represented calcification or non-hemorrhagic iron deposits, were disregarded. CMBs were categorized into 1 of 3 locations: lobar (cortical or subcortical gray), deep (deep gray matter; basal ganglia and thalamus; the white matter of the corpus callosum, internal, external, extreme capsule, brain stem and cerebellum), and mixed type [15]. The number of CMBs was recorded for each subject, and the severity of the CMBs was classified into 3 grades: in which 0 = absent, 1 = with 1 lesion, 2 = the number of CMBs ranged from 2 to 5, 3 = the number of CMBs was more than 6.

**Figure 2:**
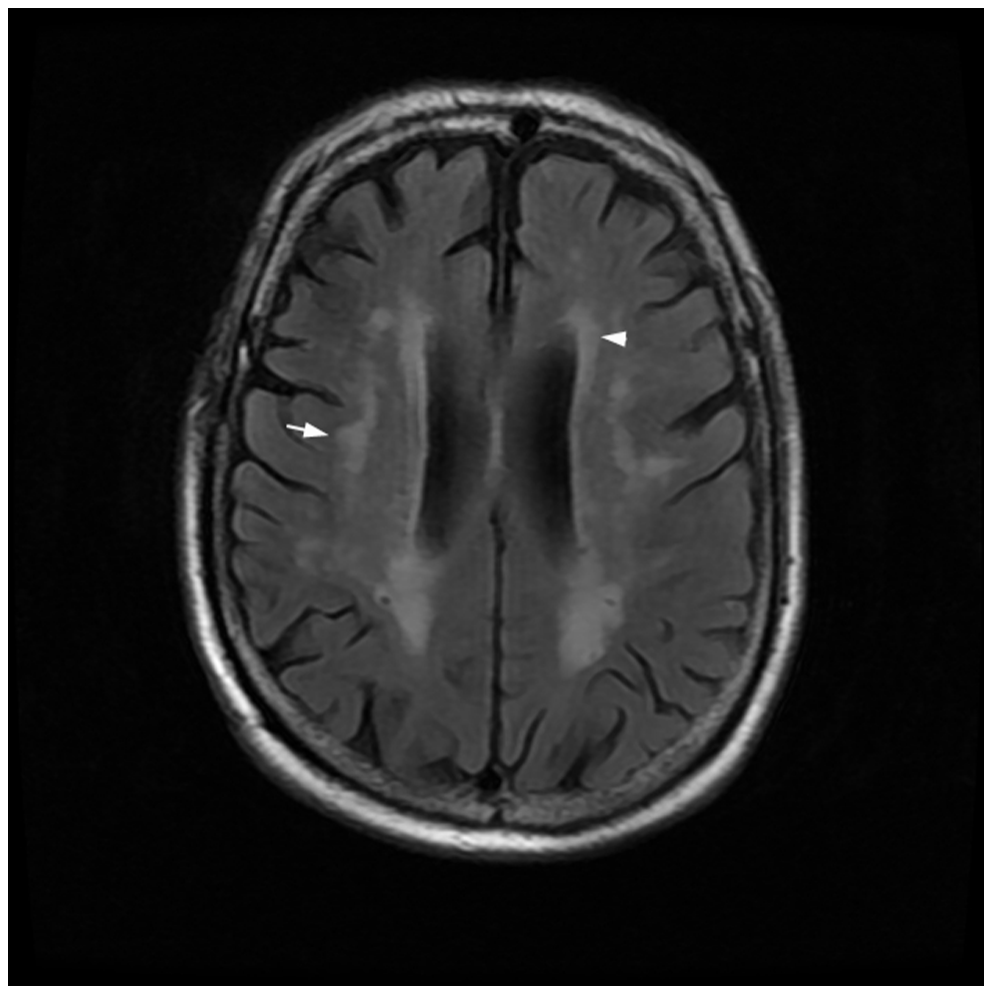
White matter hyperintensities on T2-fluid-attenuated inversion recovery sequence (T2-FLAIR). Deep white matter hyperintensities (DWMH) and peri-ventricular hyperintensities (PVH) was indicated by arrow and arrow head, respectively.

The white matter hyperintensities on the MRI was classified as PVH and DWMH, which were defined as either high intensity in the T2 -FLAIR or low intensity in the T1-FLAIR, respectively. PVH included white matter hyperintensities in contact with the ventricular wall, and DWMH included white matter hyperintensities situated in the deep white matter and separated from the ventricular wall by a strip of normal-appearing white matter (Fig. 2). The severity of the PVH and DWMH was visually rated on the T2 - FLAIR images using Fazekas’s classification [16] (ranging from 0 to 3) for PVH signals, in which 0 = absent, 1 (mild) = “caps” or pencil-thin lining, 2 (moderate) = smooth “halo”, and 3 (severe) = irregular white matter hyperintense extending into the deep white matter, and for DWMH signals, in which 0 = absent, 1 (mild) = punctuated foci, 2 (moderate) = beginning confluence of foci, 3 (severe) = large confluent areas.

Lacunar infarcts are small cavities with diameters of 3–15 mm and signal intensities comparable to cerebro-spinal fluid (CSF) on T 2 - WI. They consist in focal CSF-filled cavities, often surrounded by a hyperintense rim on T2 - FLAIR.

The MR images were independently evaluated by two of the authors of this study who were blind to the patients’ clinical profiles.

### Statistical Analysis

The data were expressed as the means ± standard deviations or proportions (%). The proportional data were analyzed using the χ^2^ test, and the continuous data were analyzed using a two-sided unpaired t-test. Logistic regression analysis was performed to examine the risk factors for asymptomatic small vessel disease (CMBs, PVH, DWMH and lacunar infarcts), and the variables included age, sex, hypertension, diabetes mellitus, smoking, alcohol consumption, hyperlipidemia and the use of anti-platelet drugs. Spearman’s correlation analysis was performed to examine the associations among CMBs, age, hypertension, and the severity of DWMH, PVH and lacunar infarcts. To exclude the effect of hypertension on the relationship between CMBs and the severity of DWMH or PVH, a hypertension-stratified correlation analysis was performed. Differences were considered significant when P<0.05. All of the statistical analyses were performed using the SPSS program, version 16.0 (SPSS, Chicago, IL, USA) for Windows XP.

## Results

### Clinical Characteristics

Of the 148 patients, there were 143 (96.6%) males and 5 (3.4%) females. The participants’ ages ranged from 61 to 96 years, and their mean age was 82.2 years. Of these patients, 113 (76.4%) patients had hypertension, 57 (38.5%) patients exhibited diabetes and 31 (20.9%) patients demonstrated hyperlipidemia. In addition, 88 (59.5%) patients were current smokers, and 45 (30.4%) patients were current drinkers. Furthermore, 119 (80.4%) patients took anti-platelet drugs. CMBs were found in 65 (43.9%) patients. The clinical characteristics of patients with different diagnoses are summarized in Table 1. Logistic regression analysis showed that age was an independent risk factor of CMBs and PVH, and hypertension was independently associated with CMBs and DWMH (Table 2). However, neither age nor hypertension was related to lacunar infarcts.

**Table 1:** Clinical Characteristics.

	ICH (n = 8)	IS (n = 63)	TIA (n = 20)	Lacunar Stroke (n = 57)
Age Mean (SD)	81.3(13.0)	84.4(6.9)	83.1(6.8)	79.6(8.7)
Male gender	8(100.0)	60(95.2)	19(95.0)	56(98.2)
Current smoker	6(75.0)	39(61.9)	11(55.0)	32(56.1)
Current drinker	1(12.5)	20(31.7)	5(25.0)	19(33.3)
History of diabetes mellitus	2(25.0)	26(41.3)	6(30.0)	23(40.4)
History of hypertension	6(75.0)	55(87.3)	13(65.0)	39(68.4)
History of hyperlipidemia	1(12.5)	10(15.9)	5(25.0)	15(26.3)
Usage of anti-platelet drugs	6(75.0)	53(84.1)	17(85.0)	43(75.4)
Presence of CMBs	6(75.0)	35(55.6)	3(15.0)	21(36.8)
Presence of PVH	4(50.0)	49(77.8)	14(70.0)	28(49.1)
Presence of DWMH	4(50.0)	53(84.1)	12(60.0)	28(49.1)

TIA = Transient ischemia stroke; ICH = Intracranial hemorrhage; IS = Ischemic stroke;

SD = Standard deviation.

**Table 2:** Risk Factors for CMBs, PVH, DWMH and LI (OR, 95% CI).

	CMBs	PVH	DWMH	LI
	OR(95%CI)	OR(95%CI)	OR(95%CI)	OR(95%CI)
Age per 10 years	1.828(1.164–2.871)	2.994(1.797–4.990)	1.404(0.916–2.152)	1.077(0.673–1.725)
Male gender	2.380(0.323–17.546)	9.785(1.130–84.744)	1.513(0.202–11.309)	0.691(0.068–6.994)
Current smoker	0.536(0.237–1.211)	0.690(0.289–1.648)	0.840(0.382–1.848)	0.828(0.354–1.938)
Current drinker	1.393(0.598–3.246)	0.889(0.369–2.143)	0.964(0.427–2.173)	1.661(0.657–4.202)
History of diabetes mellitus	1.327(0.633–2.781)	0.982(0.446–2.162)	0.743(0.362–1.522)	1.725(0.754–3.947)
History of hypertension	5.239(1.975–13.898)	2.360(0.965–5.775)	2.373(1.038–5.424)	1.897(0.787–4.574)
History of hyperlipidemia	0.564(0.227–1.400)	1.666(0.618–4.490)	1.128(0.477–2.662)	0.884(0.341–2.296)
Usage of antiplatelet drugs	0.813(0.316–2.092)	1.606(0.623–4.135)	1.404(0.916–2.152)	0.602(0.212–1.707)

CMBs = Cerebral microbleeds; PVH = Peri-ventricular hyperintensities; DWMH = Deep white matter hyperintensities; LI = Lacunar infarcts.

### Association among CMBs, WMH, Lacunar Infarcts, and Risk Factors

**Figure 3:**
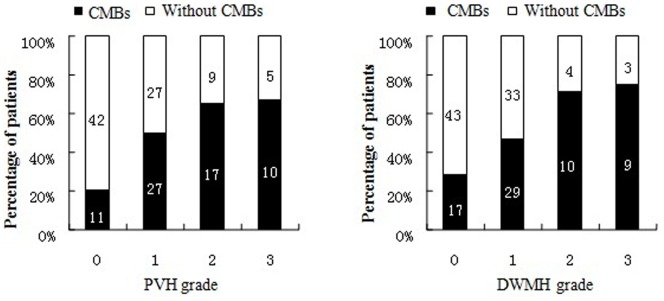
The relationship between cerebral microbleeds (CMBs) and white matter hyperintensities. The proportion of patients with CMBs increased with the severity of deep white matter hyperintensities (DWMH) (*p*<0.001, χ^2^ test) and peri-ventricular hyperintensities (PVH) (*p*<0.001, χ2 test). Each bar represents the number of patients.

The prevalence of CMBs was related to the severity of DWMH; The proportion of patients with CMBs increased from 28.3% (17/60) of patients with grade 0 DWMH to 75.0% (9/12) of patients with grade 3 DWMH (*p* = 0.002, χ^2^ test). A similar pattern was observed in the association between CMB presence and the severity of PVH (Fig. 3).

Spearman’s correlation analysis showed the severity of PVH (r = 0.347), severity of DWMH (0.314) and hypertension (0.268) had a stronger correlation with the presence of CMBs compared to age (0.166). However, no significant relationship was found between CMBs and lacunar infarcts (Table 3).

**Table 3:** Correlations amongst CMBs, WML, LI and risk factors (r).

	Age	Hypertension	CMBs	PVH	DWMH	LI
Age	1	0.003	0.166*	0.377**	0.228**	−0.006
Hypertension		1	0.268**	0.143	0.209*	0.110
CMBs			1	0.347**	0.314**	0.146
PVH				1	0.759**	0.060
DWMH					1	0.075
LI						1

CMBs = Cerebral microbleeds; PVH = Peri-ventricular hyperintensities;

DWMH = Deep white matter hyperintensities; LI = Lacunar infarcts;

*p<0.05,

**p<0.01.

### Effect of Hypertension on the Relationship between CMBs and WMH

CMBs were more frequently found in patients with hypertension (58/113, 51.3%) compared to patients without hypertension (7/35, 20.0%) (*p* = 0.001, χ^2^ test). The prevalence of DWMH was 64.6% in patients with hypertension (73/113) comparing to 42.9% (15/35) without hypertension (*p* = 0.002, χ^2^ test). However, there was no significant difference between the prevalence of PVH in patients with and without hypertension (68.1% vs. 51.4% *p* = 0.072, χ^2^ test).

To evaluate the effect of hypertension on the relationship between CMBs and WMH, correlation coefficient analyses between CMBs and PVH, and DWMH stratified by the history of hypertension were performed. Our results showed that the presence of CMBs was related to the severity of PVH in patients with (0.314) and without (0.366) hypertension. However, the presence of CMBs was associated with the severity of DWMH only in patients with hypertension (0.298) but not in patients without hypertension. Moreover, a correlation between CMBs and lacunar infarcts was not found in patients with and without hypertension (Table 4).

**Table 4:** Correlation between the presence of CMBs and PVH, DWMH stratified by hypertension(r).

	With hypertension					Without hypertension				
	Age	CMBs	PVH	DWMH	LI	Age	CMBs	PVH	DWMH	LI
Age	1	0.196*	0.429**	0.253**	0.092	1	0.059	0.183	0.132	−0.331
CMBs		1	0.314**	0.298**	0.183		1	0.366*	0.145	−0.090
PVH			1	0.778**	0.076			1	0.650**	−0.050
DWMH				1	0.065				1	0.015
LI					1					1

CMBs = Cerebral microbleeds; PVH = Peri-ventricular hyperintensities;

DWMH = Deep white matter hyperintensities; LI = Lacunar infarcts;

*p<0.05,

**p<0.01.

### Effect of CMBs Distribution on the Relationship between CMBs and WMH

To determine whether the relationship between CMBs and WMH was affected by CMB distribution, we examined the correlation between the location of CMBs and age, hypertension, PVH, DWMH and lacunar infarcts. We found that only CMBs of mixed type were associated with hypertension, PVH, and DWMH. Neither lobar CMBs nor deep CMBs showed a relationship with PVH and DWMH (Table 5).

**Table 5:** Correlation between the distribution of CMBs and presence of PVH, DWMH (%, n).

	Type of CMBs					
	NO CMBs (n = 83)	Lobar CMBs (n = 17)	Deep CMBs (n = 25)	Mixed CMBs (n = 23)	χ^2^	*P value*
PVH(+)	49.4%(41)	76.5%(13)	84.0%(21)	87.0%(20)	18.471	<0.001
DWMH(+)	48.2%(40)	58.8%(10)	72.0%(15)	87.0%(20)	13.219	0.004

CMBs = Cerebral microbleeds; PVH = Peri-ventricular hyperintensities;

DWMH = Deep white matter hyperintensities.

Interestingly, patients with PVH and DWMH exhibited a more severe grade of CMBs compared to patients without PVH and DWMH, and there was a significant correlation between the severity of CMBs and the presence of PVH and DWMH (*p*<0.001) (Table 6).

**Table 6:** Correlation between the severity of CMBs and presence of PVH, DWMH (%, n).

	Severity of CMBs					
	Grade 0 (n = 83)	Grade 1 (n = 22)	Grade 2 (n = 30)	Grade3 (n = 13)	χ^2^	*P value*
PVH(+)	49.4%(41)	77.3%(17)	80.0%(24)	100.0%(13)	20.054	<0.001
DWMH(+)	48.2%(40)	63.6%(14)	73.3%(22)	92.3%(112)	12.745	0.005

CMBs = Cerebral microbleeds; PVH = Peri-ventricular hyperintensity;

DWMH = Deep white matter hyperintensity.

## Discussion

In this study, the overall prevalence of CMBs was nearly 50%, which was higher than the prevalence in patients with ischemic strokes [5] and patients suspected of cerebrovascular disease in northeastern China [17]. The high prevalence of CMBs in this study may be attributable to the older age of our subjects and/or a higher accuracy in the detection of CMBs with a 3D T2*-weighted angiography technology. It is widely accepted that 3D T2*-weighted GRE sequence can detect more CMBs than conventional 2D GRE sequence [3].

We found a high frequency of CMBs and DWMH in patients with hypertension compared to patients without hypertension in current study. Hypertension has been identified as one of the most important risk factor for WMH and microbleeds [12], [13]. In addition, pulse wave velocity, which is a measure of arterial stiffness, had been reported that had an independent association with CMBs suggesting that hypertensive arterial stiffness may be patho-physiologically associated with CMBs [18]. Recently, Hatanaka et al [19] reported that the arterial stiffness appeared to be associated with the presence of a lacunar infarcts and WMH, independently of the risks for other cerebrovascular diseases. We confirmed a significant association between hypertension with CMBs (odds ratio 5.239) and DWMH (odds ratio 2.373). In addition, age was the most important risk factor of PVH. This outcome was consistent with findings obtained in a previous study conducted by Fazekas [20] in which the authors reported that PVH was more strongly associated with brain aging than DWMH on the basis of pathological findings. Murray and colleagues also found that DWMH was related to hypertension (*r* = 0.27) in106 non-demented subjects, and they concluded that hypertension is the principal predictors of DWMH in non-diabetic subjects [21]. Lee et al, reported that severity of PVH, not DWMH was significantly associated with incident dementia, independently of subtype of mild cognitive impairment [22]. Our data indicated that the etiologies of PVH and DWMH are different. We suggested that PVH is likely associated with aging, whereas DWMH mostly results from hypertension.

We scored DWMH and PVH separately and found that the presence of CMBs was related to the severity of DWMH and PVH. The frequency of CMBs was as high as 75% and 66.7% in patients with grade 3 of DWMH and PVH, respectively. In addition, we confirmed a close association between the number of CMBs and the severity of PVH and DWMH. A previous study, which investigated the clinical factors associated with the presence of CMBs in hemodialysis patients, showed that the presence of CMBs was significantly correlated with the presence of lacunar infarcts, PVH and DWMH [23]. In contrast, we found that age demonstrated a weaker correlation with CMBs than did PVH or DWMH and hypertension. Similar results have been reported by Yamada and colleagues [11]. Although the association of CMBs with WMH and lacunar infarcts has been investigated in different populations, to the best of our knowledge, the present study is the first study to analyze the correlation between CMBs and DWMH and PVH stratified by hypertension history. We found that CMBs were correlated with the severity of PVH independently of hypertension and were associated with the severity of DWMH only in hypertensive patients.

Lacunar infarcts are generally the consequence of hypertensive cerebral small-vessel vasculopathy. Lee et al. previously reported that the severity of microbleeds was positively correlated with the severity of lacunas and that both types of lesions were closely correlated with the degree of WMH [24]. Moreover, Igase and colleagues found that silent lacunar infarcts were strongly associated with CMBs [25]. Recently, an update of the Rotterdam Scan Study showed that lacunar infarcts were strongly associated with the presence of deep or infratentorial microbleeds but not with microbleeds in a lobar region [26]. Although the association between CMBs and lacunar infarcts has been reported in previous studies, we did not find a significant relationship between CMBs and lacunar infarcts based on current data. The probable reason for the contrary result was the difference of subjects between current and previous study. We recruited patients with acute cerebrovascular disease, but Lee focused on patients with hypertension, and the other two studies collected data from healthy subjects. Our finding implied that the existing of relationship between CMBs and lacunar infarcts was dependent on the kind of selected subjects.

It is widely accepted that different patterns of microbleed distributions are associated with different mechanisms; for example, CMBs in the deep white matter, such as the basal ganglia, thalamus, brainstem, and cerebellum, are typically associated with hypertensive vasculopathy, whereas a lobar distribution is associated with cerebral amyloid angiopathy [27]. Chowdhury et al. showed that white matter lesions and cortical microbleeds were associated with each other in healthy elderly subjects, and these changes were affected by the aging process independent of any cerebrovascular risk factors [10]. Recently, Liu et al [28] reported that systolic blood pressure variability was an independent risk factor for deep and infratentorial CMB progression. In our study, only mixed-type CMBs were associated with hypertension, PVH, and DWMH. Neither lobar CMBs nor deep CMBs showed a relationship with PVH and DWMH. These results may be due to the small number of patients with different CMB locations after stratification. Furthermore, the subjects in our study were all patients with cerebrovascular disease, and most of these patients also had hypertension. However, the occurrence and severity of CMBs were significantly associated with WMH, which suggests that these phenomena share an underlying pathological mechanism in patients with cerebral small vessel disease.

This present study has several limitations. A relatively small sample size was one limitation of this study. The study population was also small for stratified analyses. Gender bias was inevitable, because all of the subjects were recruited from a pool of retired military officers with high ranks. As a result, the majority of our subjects were males; there were only 5 females in our study. Even though, we believed this gender bias did not influence our results, because we found that gender did not influence the prevalence of CMBs not only in health subjects [5], [27] but also in patients with ischemic stroke or TIA by reviewing the related literatures [29]. In addition, we detected microbleeds with 1.5T magnetic field strength, since only 1.5 T scanners were available for us until 2012. To our knowledge, magnetic field strength is one of MRI parameters with the greatest effect on detection of CMBs. Increased magnetic field strength, to 3 T or higher, seems to improve CMBs conspicuity [30], [31]. The most critical limitation of this study was that the design was a retrospective study that did not prospectively investigate the association between CMBs and the development of WMH.

In conclusion, PVH, DWMH and hypertension were closely associated with CMBs. Hypertension determined the association between CMBs and DWMH. In addition, PVH was associated with CMBs independently of hypertension.
